# Upconversion Fluorescence Nanoprobe-Based FRET for the Sensitive Determination of *Shigella*

**DOI:** 10.3390/bios12100795

**Published:** 2022-09-27

**Authors:** Min Chen, Zhongyu Yan, Lu Han, Dandan Zhou, Yan Wang, Leiqing Pan, Kang Tu

**Affiliations:** 1 College of Food Science and Technology, Nanjing Agricultural University, Nanjing 210095, China; 2019208017@njau.edu.cn (M.C.); 2021808103@njau.edu.cn (Z.Y.); hanlu@njau.edu.cn (L.H.); 2020208021@stu.njau.edu.cn (Y.W.); pan_leiqing@njau.edu.cn (L.P.); 2 College of Light Industry and Food Engineering, Nanjing Forestry University, Nanjing 210037, China; dandanz@njfu.edu.cn

**Keywords:** *Shigella*, nanoprobe, UCNPs, GNPs, FRET

## Abstract

*Shigella* as a typical foodborne pathogen has strong survivability in the environment or food, leading to infectious diseases, yet its rapid detection technology with high selectivity and sensitivity remains challenging. In this study, complementary strand modified upconversion nanoparticles (UCNPs) can offer stable yellow-green fluorescence at 500–700 nm excited by a 980 nm laser. Importantly, *Shigella* aptamer modified gold nanoparticles (GNPs) formed by “Au−S” bond act as a fluorescence resonance energy transfer (FRET) donor and recognition element that can bind specifically to *Shigella* and significantly quench the fluorescence of complementary strand modified UCNPs. As a result, the fluorescence of our developed nanoprobe increased linearly with the increase in *Shigella* in a wide range from 1.2 × 10^2^ to 1.2 × 10^8^ CFU/mL and the detection limit was as low as 30 CFU/mL. Moreover, the fabricated upconversion fluorescence nanoprobe can achieve *Shigella* detection in contaminated chicken without enrichment in 1 h.

## 1. Introduction

Foodborne pathogens continue to be a serious public health problem throughout the world [1]. Among these foodborne pathogens, *Shigella*-contaminated food is also a cause of inflammatory diarrhea and dysentery disease. *Shigella*, as a Gram-negative bacterium, can survive at infectives in many types of foods such as meat products, fruits, vegetables, high salt/low pH food products, and packaged foods due to its acid and salt resistance properties [2,3,4]. According to the World Health Organization (WHO), more than 165 million cases of *Shigella* infection occur each year, including 1.1 million deaths. In addition, 69% of the deaths were in children under the age of five [5]. Consequently, the contamination of food-borne pathogens has introduced a serious challenge to world’s public health. Rapid, ultra-sensitive, and reliable foodborne pathogens detection technology at an early stage plays an important role in the prevention of food-borne disease outbreaks.

Direct foodborne pathogens detecting strategies without a time-consuming culture process are urgently desired [6]. Traditional foodborne pathogens’ detection methods include staining culture, DNA amplification technology, and immunoassay technology. Staining culture is a microbial culture which can only be used to distinguish Gram+ (G+) and Gram- (G-) and requires long culture time (24–72 h). DNA amplification technology including polymerase chain reaction (PCR) [7] and loop-mediated amplification (LAMP) [8] can achieve bacterial detection with high sensitivity and selectivity. However, the sample preparation of these DNA amplification detection technologies is cumbersome, and the detection results are prone to false positives. Enzyme-linked immunosorbent assay (ELISA) [9,10]) is an immunoassay technology based on antigen–antibody which can effectively eliminate the effect of false positives while the complex and strict operation procedure greatly limits the application of this method for the determination of food-borne pathogens. Hence, it is crucial to develop an accurate, rapid, highly sensitive and simple approach for the detection of foodborne pathogens.

The fluorescent probe as a promising and alternative bacterial detection method has received more and more attention due to its high sensitivity, high selectivity, and short response time [11,12]. Traditional fluorescent probes are mainly composed of some fluorescent dyes [13], fluorescent conjugated polymer [14], and quantum dot [15]. These fluorescent materials are basically converted from high-energy photons to low-energy photons, which is inevitable in the application of the fluorescent probe photobleaching phenomenon. The excitation band of these fluorescent materials is close to the fluorescence emission band, which leads to the interference of the background. In addition, these fluorescent materials have a certain degree of toxicity or some highly toxic chemicals are produced in their synthesis process. Fluorescent probes constructed from the above fluorescent materials have the problems of low target labeling efficiency, unstable fluorescent signal, compromised accuracy, and sensitivity. To get over these issues, some new fluorescence probes based on lanthanide-doped upconversion nanoparticles (UCNPs) fluorescence materials were fabricated to detect foodborne pathogens. Diametrically opposed to the traditional fluorescence materials, UCNPs have the following advantages [16,17,18,19,20,21]: (1) easy functionalization by some biologically active substances; (2) multiple fluorescence emission peaks can be obtained by doping with different rare earth elements and a high anti-Stoke shift; (3) innocuity, high resistance to photobleaching, long fluorescence lifetime and low auto-fluorescence background; and (4) ability to convert low-energy photons (such as 980 nm excitation light) into high-energy photons (such as yellow-green fluorescence). The above advantages make the fluorescence probe constructed by UCNPs display high sensitivity, simple operation, and high accuracy in food safety detection.

Recently, the fluorescent probes constructed from a series of functionalized upconversion fluorescent nanoparticles were reported to determine a variety of harmful substances (pesticide mimic diethyl chlorophosphite [22], heavy metal ions [23,24], bisulfite [25], Sudan I–IV [26], and ampicillin [27]) with high sensitivity and high selectivity. In addition, some novel upconversion fluorescent probes have been developed for the detection of foodborne pathogens in food. For instance, the UCNPs−WS2 fluorescence probe based on the fluorescence resonance energy transfer (FRET) was fabricated to detect *Escherichia coli* (*E. coli*) in water and tea powder samples with high sensitivity (LOD =17 CFU/mL) and high accuracy (96.5–101.6%) [28]; a sandwich upconversion fluorescence probe based on an inner filter (IFE) for *Listeria monocytogenes* (*L. monocytogenes*) in ham samples was reported with a low detection limit (2.8 × 10^2^ CFU/mL) and high selectivity [16]; a UCNPs−MTX PCR fluorescence probe based on IFE was developed to detect *Salmonella typhimurium* (*S. typhimurium*) DNA in chicken samples with high sensitivity (11 CFU/mL) and high selectivity. The above upconversion fluorescent probes have shown excellent performance in the detection of pathogenic bacteria in food. However, upconversion fluorescent probes for *Shigella* detection are rarely reported. Thus, developing a fluorescence probe for *Shigella* detection based UCNPs is important. 

**Scheme 1:**
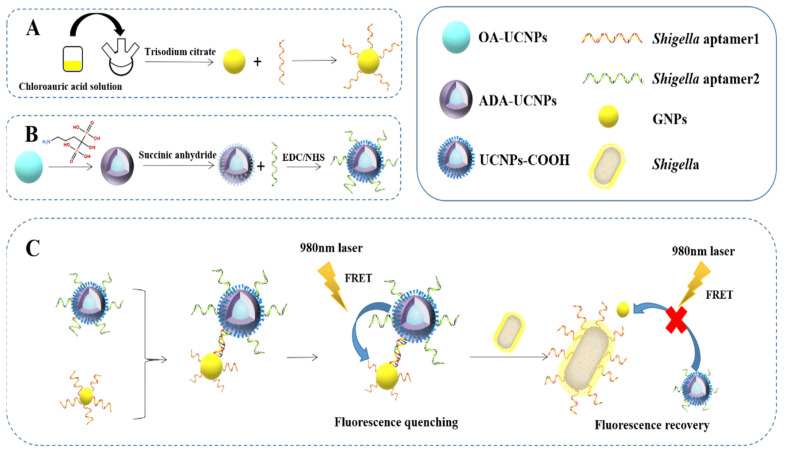
Schematic description of GNPs−UCNPs fluorescence nanoprobe for detection of *Shigella*.

Here, an upconversion fluorescence probe based on FRET between aptamer1-modification GNPs and complementary strand modified UCNPs has been designed to determinate *Shigella* in chicken samples. Scheme 1 illustrates the process of the proposed UCNPs−GNPs fluorescence probe for *Shigella* detection. First, the UCNPs−GNPs fluorescence probe was proved to be successfully fabricated by a series of characterizations (transmission electron microscopy (TEM), X-ray diffraction (XRD) spectra, FT-IR spectra). Then, we acquired fluorescence lifetimes spectra, UV-Vis, and fluorescence spectra of UCNPs−GNPs to verify the FRET mechanism. Afterwards, the upconversion fluorescence spectra of different components were obtained to confirm the feasibility of the detection method. The spectra of the UCNPs−GNPs probe with the addition of a series of different concentrations of *Shigella* and other non-target bacteria were obtained to analyze the sensitivity and selectivity of the constructed probe. Finally, we used the constructed probe to analyze the accuracy of the method by using the method of spike recovery. 

## 2. Materials and Methods

### 2.1. Materials and Chemicals

All rare earth chloride hexahydrates (YCl_3_·6H_2_O, ErCl_3_·6H_2_O, GdCl_3_·6H_2_O and YbCl_3_·6H_2_O) obtained from Sigma-Aldrich are 99% pure. Ammonium fluoride (NH_4_F), sodium hydroxide (NaOH), alendronic acid (ADA), 1-(3-Dimethylaminopropyl)-3-ethylcarbodiimide hydrochloride (EDC), hydrogen tetrachloroaurate hydrate (HAuCl_4_·3H_2_O), N-Hydroxysuccinimide (NHS), succinic anhydride, and other reagents were purchased from Alfa Aesar (Shanghai, China). All aptamers were obtained from Sangon Biotechnology Ltd. (Shanghai, China). The sequence of *Shigella* aptamer is as follows: 5′-SH-CCG GAC TAG GGC TGG TTA GCT TCA ATA CTG CTG GGC GAG G-3′ (apt1) and 5′-NH_2_-GGC CTG ATC CCG ACC AAT CGA AGT TAT GAC GAC CCG CTC C-3′ (apt2). All foodborne pathogens used in the experiment were supplied by Nanjing Agricultural University.

### 2.2. Characterization

Transmission electron microscopy (TEM, 200 kV) images were acquired on a Tecnai G2 F30. The zeta potentials of ADA−UCNPs, UCNPs−COOH, apt2−UCNPs, GNPs, and apt1−GNPs materials were measured in a neutral water solution with a Malvern Zeta sizer Nano (Malvern Instruments Ltd., Malvern, UK). Siemens D5005 instrument was used to observe the crystal structure of UCNPs. Ultraviolet–Visible (UV–Vis) absorbance spectra were recorded on a UV-1800 (Jindao, Thermofisher Co., Tokyo, Japan). Fourier transform infrared (FTIR) spectra were collected by a Nicolet IR200 FTIR spectrometer (Nicolet Co., Madison, WI, USA). Upconversion fluorescence spectra were obtained by the assembled upconversion fluorescence detection system.

### 2.3. Synthesis of apt1−GNPs

The GNPs were fabricated according to the previous literature with appropriate modifications [29]. A mixed solution of HNO_3_/HCl (volume ratio = 1:3) was used to wash all glassware used in the production of synthesis of the GNPs. HAuCl_4_ (16.98 mg) was mixed in a 250 mL flask containing 50 mL of deionized water. Then, the mixture was heated to 100 °C in a water bath under vigorous stirring. Subsequently, 5 mL of trisodium citrate solution (1%) were added to the above mixture. Then, the mixture was boiled in a water bath under vigorous stirring for 10 min. Finally, the yield GNPs were cooled down to room temperature and stored at 4 °C in the refrigerator. The apt1−GNPs were synthesized by a previously reported method with appropriate modifications [29]. A total of 1 mL of 100 nM apt1 was mixed into 13 mL of GNPs solution and reacted under vigorous stirring under room temperature for 30 min. Finally, the yield apt1−GNPs were stored at 4 °C in the refrigerator.

### 2.4. Fabrication of apt2−UCNPs

Oleic acid (OA)-capped NaYF_4_: Yb, Er UCNPs was fabricated using a reported solvothermal method [30]. RECl_3_·6H_2_O (0.8 mmol, Gd:Y:Er:Yb = 0.3:0.48:0.02:0.2) dissolved in 4 mL of methanol was added to a 250 mL three-necked round-bottom flask containing 14 mL of 1-octadecene and 6 mL of oleic acid. Next, the above mixture solution was heated to 160 °C under a nitrogen atmosphere and vigorous stirring for 30 min. After the reaction, the mixture solution was cooled down to 50 °C and added dropwise 20 mL of the mixed methanol solution (2.5 mmoL NaOH and 4 mmoL NH_4_F) under vigorous stirring, and then the mixture solution was transferred to a water bath at 50 °C and kept reacting for 40 min. Subsequently, the mixture was heated to 70 °C for 50 min to evaporate methanol. Finally, the mixture solution was heated to 300 °C in a nitrogen atmosphere and vigorously stirred for 60 min. After the reaction, the mixture solution was cooled down to room temperature. The yield OA−UCNPs were washed three times with cyclohexane-ethanol and dried in a vacuum oven at 55 °C for 8 h.

Alendronic acid-modified upconversion nanoparticles (ADA−UCNPs) were synthesized by a previously reported method with appropriate modifications and the detailed procedure was as follows [18]: 200 mg OA−UCNPs were dispersed in 8 mL of CHCl_3_ by sonication for 30 min. Next, the UCNPs solution was transferred to a 100 mL centrifuge tube containing 4 mL of ethanol and 6 mL of 8.3 mg/ mL ADA solution. Then, the mixture was adjusted to pH 2–3 using a 1M HCl solution and kept reacting for 1 h. Finally, the yield ADA−UCNPs were washed three times with deionized water–ethanol (1:1) and dried in the vacuum oven at 60 °C overnight. 

Carboxyl-modified upconversion (UCNPs−COOH) was prepared according to the previous literature with appropriate modifications. Firstly, 200 mg ADA−UCNPs were dispersed in 15 mL methylbenzene by sonication for 30 min, then 5 mL of succinic anhydride (0.16 M) solution were mixed into the ADA−UCNPs solution. Then, the mixture was heated to 80 °C in a nitrogen atmosphere and vigorously stirred for 12 h. After the reaction, the mixture solution was cooled down to 35 °C and washed three times with deionized water. Finally, the yield UCNPs−COOH was dried in a vacuum oven at 60 °C overnight.

The procedure of aptamer-functionalized UCNPs was adapted from a previously reported method with minor modification [31]. Firstly, 5 mg of UCNPs−COOH were dispersed in 2.5 mL MES buffer (50 mM, pH = 6) by sonication for 30 min. Next, 80 mL of mixed solution (2 mg/mL EDC and 2 mg/mL NHS) were added to the UCNPs−COOH solution and reacted for 2 h under stirring. Then, the UCNPs−COOH was washed three times with PBS buffer solution (pH = 7.4) and re dispersed in 2.5 mL PBS buffer solution (pH = 7.4). Then, 500 μL of 100 apt2 were added to the UCNPs−COOH solution and reacted for 2 h under stirring at room temperature. Finally, the yield apt2 modification UCNPs was washed with a PBS buffer solution.

### 2.5. Bacteria Culture

All bacterial strains used in this experiment were *Shigella* (ATCC 12022), *E. coli* (ATCC 43889), *S. typhimurium* (ATCC 14028), *L. monocytogenes* (ATCC ATCC19111), *Staphylococcus aureus* (*S. aureus*, ATCC 130), *Pseudomonas aeruginosa* (*P. aeruginosa*, ATCC 27853), and *Streptococcus thermophila* (*S. thermophila,* ATCC03872). *Shigella* (ATCC 12022) as target bacteria was detected using the fabricated upconversion fluorescence probe. Meanwhile, *S. typhimurium* (ATCC 14028), *L. monocytogenes* (ATCC ATCC19111), *S. aureus* (ATCC 130), *P. aeruginosa* (ATCC 27853), and *S. thermophila* (ATCC 03872) as control groups were selected to confirm the specificity of an upconversion fluorescence probe. All bacteria used in this research work was cultured in a Luria–Bertani (LB) broth at 37 °C overnight. The bacterial cells were washed three times with deionized water under centrifugation (8000 rpm). The plate counting was used to calculate the concentration of the bacteria.

### 2.6. Construction of Upconversion Fluorescent Probe and Detection of Shigella 

Firstly, we used plate counts to calculate the cell concentration of *Shigella* (1.2 × 10^8^ CFU/mL), and the experimental groups were prepared with the *Shigella* suspension concentration gradients of 1.2 × 10^1^, 1.2 × 10^2^,1.2 × 10^3^, 1.2 × 10^4^, 1.2 × 10^5^, 1.2 × 10^6^, 1.2 × 10^7^ and 1.2 × 10^8^ CFU/mL. The upconversion fluorescent probe was fabricated as follows: a total of 140 μL of a series of concentrations of *Shigella* were added to 140 μL of 100 nM apt1−GNPs and reacted for 25 min; then, 20 μL of 4 mg/mL apt2−UCNPs were added to the above mixture and continued reacting for 11 min. Finally, the mixture solution was detected using the assembled upconversion fluorescence detection system.

### 2.7. Recovery Experiments for Samples

Chicken samples were purchased from a local supermarket in Nanjing, China. In order to eliminate the interference of target bacteria or other non-target bacteria in the chicken samples as much as possible, we used sterile saline to wash chicken samples (25 g) and exposed them to a 30 W UV lamp for 30 min. Then, the concentration range (10^3^–10^5^ CFU/mL) of *Shigella* was spiked in chicken samples. The detailed procedure of *Shigella* detection in the chicken samples was as follows: Firstly, the macroaggregates in chicken sample solution were removed by standing for 30 min. Then, the 0.45 μm filtration membrane was used to filter the chicken samples’ supernatant. Finally, the filtrate solution was determined by the developed upconversion fluorescence probe and the traditional plate counting method, respectively.

## 3. Results and Discussion

### 3.1. Characterization

**Figure 1:**
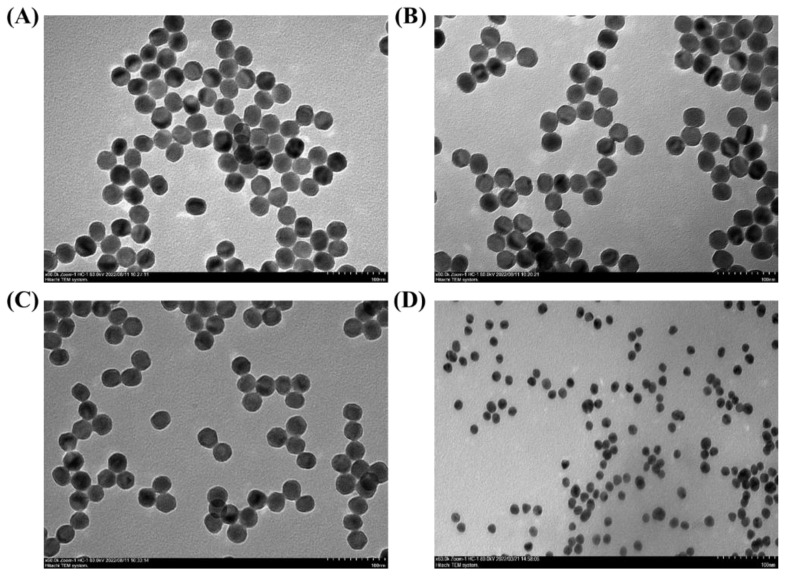
TEM image of OA−UCNPs (**A**), ADA−UCNPs (**B**), UCNPs−COOH (**C**), and GNPs (**D**).

**Figure 2:**
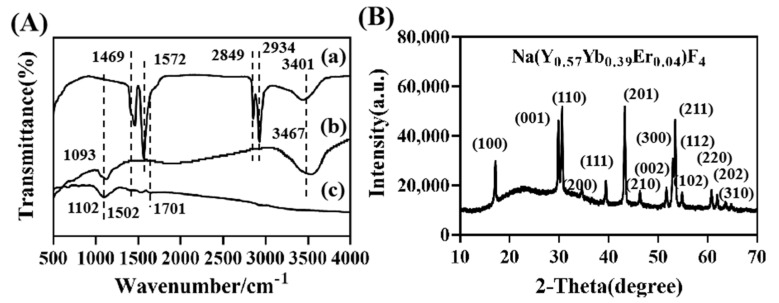
FTIR spectra of OA−UCNPs (a), ADA−UCNPs (b), UCNPs−COOH (c) (**A**); XRD patterns of UCNPs (**B**).

TEM characterizations of OA−UCNPs, ADA−UCNPs, UCNPs−COOH, and GNPs were displayed in Figure 1. The images (Figure 1A) revealed that OA−UCNPs have a uniform size with shaped particles (35 nm). As seen in Figure 1B,C, the shape of UCNPs after ADA and carboxyl modification has no obvious change. As shown in Figure 1D, the particle diameter of GNPs was 20 nm. The surface group of the UCNP after ADA and carboxyl modification was confirmed by FT-IR spectroscopy. As shown in Figure 2A, five stretching vibration bands at 1469 cm^−1^, 1572 cm^−1^, 2849 cm^−1^, 2934 cm^−1^, and 3401 cm^−1^ were found, and these bands belonged to the =C-H, COO- and -CH-stretching vibration, respectively (curve a) [32]. After surface modification with ADA, the four bands at around 1469 cm^−1^, 1572 cm^−1^, 2849cm^−1^, and 2934 cm^−1^ disappeared, and a new stretching vibration band at 1093 cm^−1^ appeared, which was associated with the NH_2_-stretching vibration, validating successful modification of ADA (curve b) [33]. After surface modification with carboxyl, the band at around 3467 cm^−1^ disappeared, and two new stretching vibration bands at 1502 cm^−1^ and 1701cm^−1^ appeared. This is because of the successful graft of COOH groups onto NH_2_- groups (curve c) [32]. Hence, the above results further proved that the UCNPs−COOH composites were successfully modified. The XRD measurements of upconversion nanoparticles powder were used to confirm the structure of the as-prepared OA−UCNPs, and the results were shown in Figure 2B. The diffraction peaks of the OA−UCNPs could be well matched with the standard JCPDS: 28-1192 card, confirming the tetragonal phase of the Na(Y_0.57_Yb_0.39_Er_0.04_) YF_4_ material.

### 3.2. Feasibility of the Upconversion Fluorescence Probe

**Figure 3:**
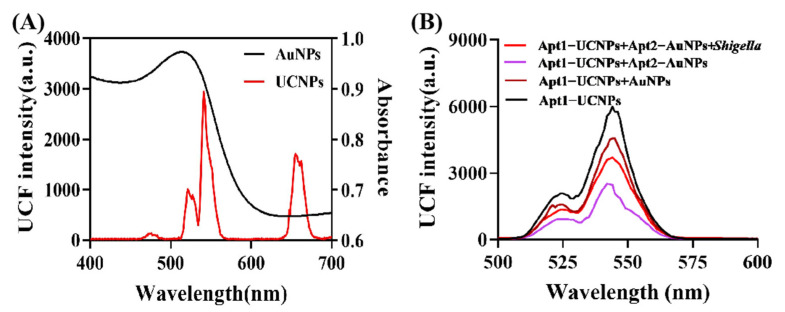
Fluorescence spectra of UCNPs and UV-Vis absorption spectra of GNPs (**A**); fluorescence spectra of apt2−UCNPs with addition of GNPs, apt1−GNPs, and apt1−GNPs−*Shigella* (**B**).

**Figure 4:**
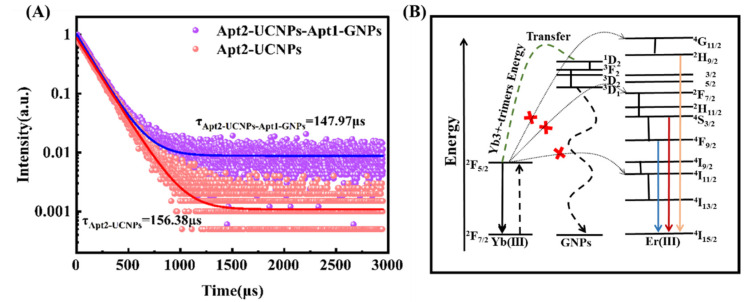
Fluorescence lifetimes spectra of apt2−UCNPs and apt1−GNPs−apt2−UCNPs (**A**); plausible energy transfer mechanism from Na(Y0_.57_Yb_0.39_Er_0.04_) YF_4_ to GNPs (**B**).

The detection of *Shigella* relies on a FRET process between apt2−UCNPs acting as a fluorescence donor and apt1−GNPs acting as a fluorescence acceptor and target bacterial recognition element. As demonstrated in Scheme 1, the specific *Shigella* aptamer modification GNPs were fabricated by the bond of “Au−S”. OA−UCNPs is sequentially modified by alendronate and carboxyl groups to generate UCNPs−COOH, and the complementary strand was conjugated on UCNPs−COOH. In the absence of *Shigella*, the UCNPs−GNPs complex formed by the complementary pairing of aptamer, causing the distance between GNPs and UCNPs to be less than 10 nm, led to the decreasing fluorescence. Upon addition of *Shigella*, *Shigella* was captured by apt1 and fell from the surface of GNPs. The bare gold nanoparticles cannot be adsorbed on the upconversion surface by base complementary pairing, leading to fluorescence recovering. To verify the FRET mechanism between apt2−UCNPs and apt1−GNPs, the upconversion fluorescence spectra and absorption spectra of the mixed UCNPs−GNPs were tested. As seen in Figure 3A and Figure 4B, the emission peaks of OA−UCNPs were located at 525, 545, and 650 nm, which were attributed to the 2H_9/2_ → 4F_15/2_, 4S_3/2_ → 4F_15/2_, and 4I_11/2_ → 4F_15/2_ transitions of Er^3+^, respectively [28]. The GNPs show a wide absorption band at 400–700 nm, which is overlapped with the emission peak of OA−UCNPs at 525, 545, and 650 nm. In addition, the distance between apt2−GNPs and apt1−UCNPs formed is less than 10 nm due to the complementary pairing of aptamer, led to the occurrence of FRET [32]. In order to further analyze the mechanism of fluorescence quenching, the fluorescence lifetimes of apt1−UCNPs and apt1−UCNPs−apt2−GNPs were determined, and the results are showed in Figure 4A. After incubation with the apt1−GNPs, the fluorescence lifetime of apt2−UCNPs declined significantly with the τ decreasing from 156.38 μs to 147.97 μs. Thus, FRET was proved to mainly contribute to the fluorescence quenching of apt2−UCNPs by apt1−GNPs.

### 3.3. Characterization of the Construction Process of Upconversion Fluorescence Probe

**Figure 5:**
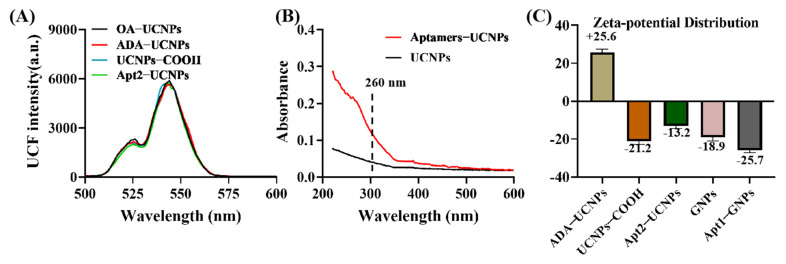
UV-Vis spectra of the UCNPs−COOH before and after aptamers modification (**A**); fluorescence spectra of OA−UCNPs, ADA−UCNPs, UCNPs−COOH, and apt1−UCNPs (**B**); zeta-potential of ADA−UCNPs, UCNPs−COOH and apt2−UCNPs, GNPs, and apt1−GNPs (**C**).

The fluorescence spectra of OA−UCNPs, ADA−UCNPs, UCNPs−COOH, and apt1−UCNPs at the same concentration were determined to confirm the stability of upconversion fluorescence. As seen in Figure 5A, it can be found that the fluorescence spectra after ADA modification, carboxyl modification, and aptamer modification hardly changed significantly, which confirmed that the fluorescence of upconversion nanoparticles has good stability. The aptamer modification UCNPs was confirmed from the results of UV-Vis absorption spectra (Figure 5B), and the new absorption peak at 260 nm appeared after aptamer modification, verifying the formation of apt2 modification UCNPs. In addition, the zeta potential of ADA−UCNPs, UCNPs−COOH, apt2−UCNPs, GNPs, and apt1−GNPs were determined, and the results are shown in Figure 5C, ADA−UCNPs have positive charge with zeta potential of 25.6 mV. After carboxyl modification, the zeta potential of UCNPs positively shifts to −21.2 mV, revealing that the -COOH groups are successfully modified on the surface of upconversion nanoparticles. Upon conjugation of apt2, the zeta potential changed into −13.2 mV, which is because of the positive charge of aptamer. Bare GNPs are negatively-charged with zeta potential of −18.9 mV. After *Shigella* apt1 modification, the zeta potential of GNPs changes into −25.7 mV, revealing that the bare GNPs (−18.9 mV) or apt1−GNPs cannot be adsorbed on apt2−UCNPs (−13.2 mV) by electrostatic adsorption. In addition, the fluorescence spectra of apt1−UCNPs with the addition of GNPs, apt1−GNPs, and apt1−GNPs−*Shigella* were tested to confirm the feasibility of the upconversion fluorescence probe for detection of *Shigella*. As shown in Figure 3B, the upconversion fluorescence intensity at 545 nm after the addition of bare GNPs is decreased due to IFE between apt2−UCNPs and GNPs. After incubation of apt1−GNPs, the upconversion fluorescence intensity at 545 nm is significantly lower than UCNPs−GNPs, which is because of the FRET between apt1−UCNPs and apt2−GNPs. In addition, the upconversion fluorescence intensity of apt1−UCNPs−apt2−GNPs with the addition of *Shigella* occurred for recovery. Based on the above fluorescence changes, the developed upconversion fluorescence probe can be used to detect *Shigella*. 

### 3.4. Optimization of Experimental Conditions

**Figure 6:**
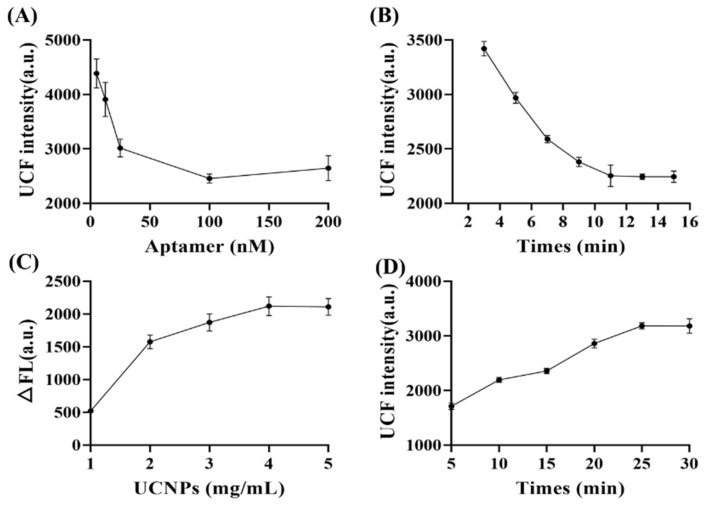
Optimized condition of apt1 (**A**), the incubation time of apt1−GNPs (**B**), apt2−UCNPs (**C**), and the incubation time between the probe and *Shigella* (**D**).

To obtain high performance of the developed probe, this experiment optimized the following related parameters: (A) the concentration of apt1, (B) the incubation time of apt1−GNPs, (C) the concentration of apt2−UCNPs, and (D) the incubation time between the nanoprobe and *Shigella*. Firstly, the fluorescence intensity at 545 nm was significantly affected by the concentration of apt1 when the concentration of apt2−UCNPs was fixed. The upconversion intensity at 545 nm successively decreases as the concentration of apt1 increases, and the fluorescence intensity tended to be stable when the apt1 was 100 nM (Figure 6A). The reaction time can affect a fluorescence quenching effect of apt1−GNPs on apt2−UCNPs. When the reaction time is 11 min, the upconversion fluorescence intensity reached the lowest point. Thus, the optimal reaction time between apt2−UCNPs and apt1−GNPs was 11 min in the subsequent experiments (Figure 6B). The addition ratio of apt2−UCNPs and apt1−GNPs has a great influence on the *Shigella* detection results. With the increased concentration of apt2−UCNPs, the fluorescence response (ΔFL) increased, and the ΔFL remained stable after 4 mg/mL. Thus, 4 mg/mL was selected as the best concentration (Figure 6C). The incubation between apt1−GNPs and *Shigella* has a great impact on the selectivity of the developed upconversion fluorescence probe. The upconversion intensity at 545 nm successively increases as the incubation time increases, and the fluorescence intensity tended to be stable when the times reached 25 min. Therefore, 25 min were selected as the best incubation time (Figure 6D).

### 3.5. Determination of Shigella

**Figure 7:**
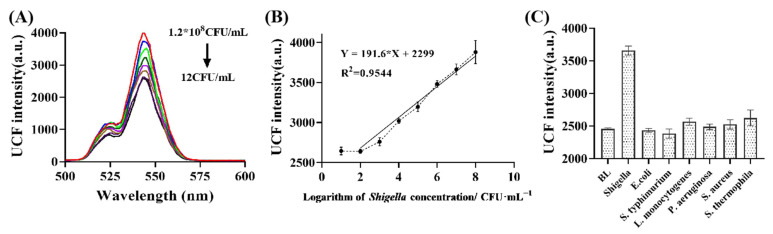
Fluorescence spectra of the nanoprobe at various concentrations of *Shigella* (**A**); linear relationship between the fluorescence at 550 nm versus *Shigella* concentration (**B**); selectivity of the developed nanoprobe (**C**).

Under the optimized conditions, the fabricated upconversion fluorescence probe was used to detect *Shigella*. As seen in Figure 7A, the upconversion intensity at 545 nm increased with the increased concentration of *Shigella*. This phenomenon was ascribed to the apt1 firmly adhered to the surface of bacteria, and the GNPs fell from the surface of apt−UCNPs. The higher the *Shigella* concentration, the more GNPs fell from the apt2−UNCPs surface; thus, a higher fluorescence signal can be acquired. A linear relationship was obtained between the upconversion intensity at 545 nm and the *Shigella* concentration. Density ranged from 1.2 × 10^2^ to 1.2 × 10^8^ CFU/mL (Figure 7B). The linear equation was Y = 2299 + 191.6 X, with R^2^ = 0.9544 (Figure 2B). The limit of detection (LOD) of the fabricated upconversion fluorescence probe was determined to be 30 CFU/mL, based on the formula of LOD = 3 Sa/S_b_ (3 is the factor at the 99% confidence level, Sa is the standard deviation of 10 blank upconversion fluorescence measurements, and S_b_ is the slope of the *Shigella* detection calibration plot). Furthermore, in comparison with the linear range, LOD and detection time of the developed upconversion fluorescence probe with other reported methods [33,34,35,36,37,38] confirmed that the probe can achieve *Shigella* detection with high sensitivity and meets the requirements for contaminated sample detection (Table 1).

### 3.6. Selectivity of the Developed Upconversion Fluorescence Probe

In order to evaluate the specificity of this developed upconversion fluorescence probe for *Shigella*, we added other bacteria (e.g., *Shigella* (ATCC 12022), *E. coli* (ATCC 43889), *S. typhimurium* (ATCC 14028), *L. monocytogenes* (ATCC 19111), *S. aureus* (ATCC 130), *P. aeruginosa* (ATCC 27853), and *S. thermophila* (ATCC 03872)) into the UCNPs−GNP probe. As shown in Figure 7C, we observed that only *Shigella* (ATCC 12022) induced a dramatic fluorescence recovering at 545 nm. In addition, we tested the fluorescence intensity of our developed probe with the addition of other non-target bacterial and found that *E. coli* (ATCC 43889), *S. typhimurium* (ATCC 14028), *L. monocytogenes* (ATCC 19111), *S. aureus* (ATCC 130), *P. aeruginosa* (ATCC 27853), and *S. thermophila* (ATCC 03872) in this upconversion fluorescence probe had no significant effect on fluorescence intensity at 545 nm, which verified that the upconversion fluorescence probe had excellent specificity.

### 3.7. Analytical Application

The practicability of the proposed upconversion fluorescence probe in the analysis of chicken samples was further analyzed by spiked food samples, and the results are shown in Table 2. The recoveries in chicken samples were 86.7–113.4%, which confirmed that the developed fluorescence probe had excellent accuracy and practicability in contaminated samples’ detection.

## 4. Conclusions

A UCNPs@GNPs fluorescence probe was developed for rapid sensing of *Shigella* based on apt1 modified GNPs (fluorescence acceptor) as reorganization elements and complementary strand modified UCNPs (fluorescence donor). The formation of apt1−*Shigella* complex resulted in the recovery of upconversion fluorescence intensity at 545 nm, and *Shigella* was detected based on the increase in upconversion fluorescence at 545 nm. A linear response was calculated in the range of 1.2 × 10^2^ to 1.2 × 10^8^ CFU/mL with an LOD of 30 CFU/mL. By the analysis of the interference of non-target bacteria with the fluorescence probe, it was found that the probe had good specificity. The study also provides one concept for design and synthesis of a UCNPs−GNPs fluorescence probe for *Shigella* detection in the food safety field.

## Figures and Tables

**Table 1:** Comparison of different sensors for *Shigella* detection.

Sensors	Linear Range (CFU/mL)	LOD (CFU/mL)	Detection Time	Reference
Electrochemical sensor	3 × 10^3^–3 × 10^4^	18	78 min	[34]
wave fiber sensor	10^2^–10^4^	100	-	[35]
SERS sensor	10–10^6^	10	1.5 h	[36]
Fluorescence sensor	10^2^–10^7^	30	1 h	[38]
Fluorescence sensor	10^3^–10^7^	10^3^	1.5 h	[37]
Upconversion fluorescence sensor	1.2 × 10^2^–1.2 × 10^8^	30	1 h	This work

**Table 2:** Determination of *Shigella* in real chicken samples using the developed upconversion fluorescence probe.

Sample	Spiked Levels (CFU/mL)	Our Developed Method (Mean ^a^ ± SD ^b^) (CFU/mL)	Flat Colony Counting Method (Mean ^a^ ± SD ^b^) (CFU/mL)	Recovery (%)
Chicken	10^3^	(1.068 ± 0.170) × 10^3^	(1.077 ± 0.054) × 10^3^	106.8
	10^3^	(0.964 ± 0.084) × 10^3^	(0.977 ± 0.033) × 10^3^	96.4
	10^3^	(0.881 ± 0.021) × 10^3^	(0.903 ± 0.028) × 10^3^	88.1
	10^4^	(1.134 ± 0.160) × 10^4^	(1.090 ± 0.030) × 10^4^	113.4
	10^4^	(1.111 ± 0.082) × 10^4^	(1.127 ± 0.053) × 10^4^	111.1
	10^4^	(0.867 ± 0.026) × 10^4^	(0.933 ± 0.037) × 10^4^	86.7
	10^5^	(1.042 ± 0.032) × 10^5^	(1.093 ± 0.041) × 10^5^	104.2
	10^5^	(0.952 ± 0.010) × 10^5^	(1.003 ± 0.020) × 10^5^	95.2
	10^5^	(0.895 ± 0.047) × 10^5^	(0.970 ± 0.069) × 10^5^	89.5

^a^ Mean concentration; ^b^ Standard deviation.

## Data Availability

Not applicable.

## References

[1] Warren B.R., Parish M.E., Schneider K.R. (2006). Shigella as a Foodborne Pathogen and Current Methods for Detection in Food. Crit. Rev. Food Sci. Nutr..

[2] Levin R.E. (2009). Molecular Methods for Detecting and Discriminating Shigella Associated with Foods and Human Clinical Infec-tions—A Review. Food Biotechnol..

[3] Elahi N., Kamali M., Baghersad M.H., Amini B. (2019). A fluorescence Nano-biosensors immobilization on Iron (MNPs) and gold (AuNPs) nanoparticles for detection of *Shigella* spp.. Mater. Sci. Eng. C.

[4] Liding Z., Qiujiang W., Qinqin H., Qiang C., Wenlin T., Jinyang Z., Yuzhu S., Xueshan X. (2018). Detection of Shigella in Milk and Clinical Samples by Magnetic Immunocaptured-Loop-Mediated Isothermal Amplification Assay. Front. Microbiol..

[5] He P., Wang H., Yan Y., Zhu G., Chen Z. (2022). Development and Application of a Multiplex Fluorescent PCR for Shigella Detection and Species Identification. J. Fluoresc..

[6] Han X., Liu Y., Yin J., Yue M., Mu Y. (2021). Microfluidic devices for multiplexed detection of foodborne pathogens. Food Res. Int..

[7] Lehniger L., Rudloff A., Pollok S., Grosse N., Wessel K., Brendel M., Popp J., Weber K. (2021). A Model System for Sensitive Detection of Viable *E. coli* Bacteria Combining Direct Viability PCR and a Novel Microarray-Based Detection Approach. Chemosensors.

[8] Chen H., Li Y.K., Zhang T.T., Bi Y., Shu M., Zhong C., Tang K.J., Wu G.P. (2021). A Novel Real-Time Loop-Mediated Isothermal Amplification Combined with Immunomagnetic Beads Separation and Ethidium Bromide Monoazide Treatment for Rapid and Ultrasensitive Detection of Viable Escherichia coli O157:H7 in Milk. Food Anal. Methods.

[9] Xia J.F., Qiu S., Zeng H.J., Liu C., Liu Q. (2021). A rapid detection of Escherichia coli O157:H7 by competition visual antigen macroarray. J. Food Saf..

[10] Matsui K., Tanabe S., Sun S.Y., Nguyen D., Kinoshita F., Yamamoto Y., Shiigi H. (2020). Development of Metal Nanoparticle-immobilized Microplate for High-throughput and Highly Sensitive Fluorescence Analysis. Anal. Sci..

[11] Liu X.K., Li C.H., Wang Z., Zhang N., Feng N., Wang W.J., Xin X. (2021). Luminescent Hydrogel Based on Silver Nanocluster/Malic Acid and Its Composite Film for Highly Sensitive Detection of Fe^3+^. Gels.

[12] Wang W.J., Wang Z., Sun D., Li S.L., Deng Q.H., Xin X. (2022). Supramolecular Self-Assembly of Atomically Precise Silver Nanoclusters with Chiral Peptide for Temperature Sensing and Detection of Arginine. Nanomaterial.

[13] Wang K., Ye Y.X., Jiang C.Y., Guo M.Y., Zhu H.L. (2021). Design and synthesis of a novel “turn-on” fluorescent probe based on benzofuran-3(2H)-one for detection of hydrazine in water samples and biological systems. Dyes Pigment..

[14] Wang J., Peng R., Luo Y., Wu Q., Cui Q. (2021). Preparation of fluorescent conjugated polymer micelles with multi-color emission for latent fingerprint imaging. Colloids Surf. A Physicochem. Eng. Asp..

[15] Lu H., Cui H., Duan D., Li L., Ding Y. (2021). A novel molecularly imprinted electrochemical sensor based on a nitrogen-doped graphene oxide quantum dot and molybdenum carbide nanocomposite for indometacin determination. Analyst.

[16] Li Y., Chen M., Fan X., Peng J., Pan L.Q., Tu K., Chen Y.P. (2022). Sandwich fluorometric method for dual-role recognition of Listeria monocytogenes based on antibiotic-affinity strategy and fluorescence quenching effect. Anal. Chim. Acta.

[17] Annavaram V., Chen M., Kutsanedzie F.Y.H., Agyekum A.A., Zareef M., Ahmad W., Hassan M.M., Huanhuan L., Chen Q. (2019). Synthesis of highly fluorescent RhDCP as an ideal inner filter effect pair for the NaYF4:Yb,Er upconversion fluorescent nanoparticles to detect trace amount of Hg(II) in water and food samples. J. Photochem. Photobiol. A Chem..

[18] Chen M., Han L., Zhou D.D., Kong L.Y., Pan L.Q., Tu K. (2022). Amplified UCNPs-Mitoxantrone dihydrochloride fluorescence PCR sensor based on inner filter for ultrasensitive and rapid determination of Salmonella typhimurium. Sens. Actuators B Chem..

[19] Chen M., Kutsanedzie F.Y.H., Cheng W., Li H., Chen Q. (2019). Ratiometric fluorescence detection of Cd^2+^ and Pb^2+^ by inner filter-based upconversion nanoparticle-dithizone nanosystem. Microchem. J..

[20] Liang M.Y., Zhao B., Xiong Y., Chen W.X., Huo J.Z., Zhang F., Wang L., Li Y. (2019). A “turn-on” sensor based on MnO2 coated UCNPs for detection of alkaline phosphatase and ascorbic acid. Dalton Trans..

[21] Si F., Zou R., Jiao S., Qiao X., Guo Y., Zhu G. (2018). Inner filter effect-based homogeneous immunoassay for rapid detection of imidacloprid residue in environmental and food samples. Ecotoxicol. Environ. Saf..

[22] Wang S.L., Wang X.B., Chen X.X., Cao X.Z., Cao J., Xiong X.F., Zeng W.B. (2016). A novel upconversion luminescence turn-on nanosensor for ratiometric detection of organophosphorus pesticides. RSC Adv..

[23] Liang T., Li Z., Song D., Shen L., Zhuang Q.G., Liu Z.H. (2016). Modulating the Luminescence of Upconversion Nanoparticles with Heavy Metal Ions: A New Strategy for Probe Design. Anal. Chem..

[24] Chu Z.Y., Wang W.N., Zhang C.Y., Ruan J., Chen B.J., Xu H.M., Qian H.S. (2019). Monitoring and removal of trace heavy metal ions via fluorescence resonance energy transfer mechanism: In case of silver ions. Chem. Eng. J..

[25] Chen H.Q., Xia W.Y., Gao Q., Wang L. (2021). Sensitive quantitative image analysis of bisulfite based on near-infrared upconversion luminescence total internal reflection platform. Talanta.

[26] Fang A.J., Long Q., Wu Q.Q., Li H.T., Zhang Y.Y., Yao S.Z. (2016). Upconversion nanosensor for sensitive fluorescence detection of Sudan I-IV based on inner filter effect. Talanta.

[27] Zhang Y.L., Hassan M.M., Rong Y.W., Liu R., Li H.H., Ouyang Q., Chen Q.S. (2022). A solid-phase capture probe based on upconvertion nanoparticles and inner filter effect for the determination of ampicillin in food. Food Chem..

[28] Wang P., Wang A., Hassan M.M., Ouyang Q., Chen Q. (2020). A highly sensitive Upconversion nanoparticles-WS2 nanosheet sensing platform for Escherichia coli detection. Sens. Actuators B Chem..

[29] Chen M., Hassan M., Li H.H., Chen Q.S. (2020). Fluorometric determination of lead(II) by using aptamer-functionalized upconversion nanoparticles and magnetite-modified gold nanoparticles. Microchim. Acta.

[30] Chen M., Kutsanedzie F.Y.H., Cheng W., Agyekum A.A., Li H.H., Chen Q.S. (2018). A nanosystem composed of upconversion nanoparticles and N, N-diethyl-p-phenylenediamine for fluorimetric determination of ferric ion. Microchim. Acta.

[31] Ouyang Q., Yang Y., Ali S., Wang L., Chen Q. (2021). Upconversion nanoparticles-based FRET system for sensitive detection of Staphylococcus aureus. Spectrochim. Acta Part A Mol. Biomol. Spectrosc..

[32] Liu Y., Ouyang Q., Li H., Chen M., Zhang Z., Chen Q. (2018). Turn-on Fluoresence Sensor for Hg2+ in Food Based on FRET between Aptamers-Functionalized Upconversion Nanoparticles and Gold Nanoparticles. J. Agric. Food Chem..

[33] Chen M., Song Y.Q., Han L., Zhou D.D., Wang Y., Pan L.Q., Tu K. (2022). An Ultrasensitive Upconversion Fluorescence Aptasensor Based on Graphene Oxide Release and Magnetic Separation for Staphylococcus aureus Detection. Food Anal. Methods.

[34] Luo J.L., Wang J.P., Mathew A.S., Yau S.T. (2016). Ultrasensitive Detection of Shigella Species in Blood and Stool. Anal. Chem..

[35] Xiao R., Rong Z., Long F., Liu Q.Q. (2014). Portable evanescent wave fiber biosensor for highly sensitive detection of Shigella. Spectrochim. Acta Part A Mol. Biomol. Spectrosc..

[36] Wu S.J., Duan N., He C.X., Yu Q.R., Dai S.L., Wang Z.P. (2020). Surface-enhanced Raman spectroscopic-based aptasensor for Shigella sonnei using a dual-functional metal complex-ligated gold nanoparticles dimer. Colloids Surf. B Biointerfaces.

[37] Song M.S., Sekhon S.S., Shin W.R., Kim H.C., Min J., Ahn J.Y., Kim Y.H. (2017). Detecting and Discriminating Shigella sonnei Using an Aptamer-Based Fluorescent Biosensor Platform. Molecules.

[38] Gong W.H., Duan N., Wu S.J., Huang Y.K., Chen X.J., Wang Z.P. (2015). Selection, identification, and application of dual DNA aptamers against Shigella sonnei. Anal. Methods.

