# Correction: Chen et al. Upconversion Fluorescence Nanoprobe-Based FRET for the Sensitive Determination of *Shigella*. *Biosensors* 2022, *12*, 795

**DOI:** 10.3390/bios13070713

**Published:** 2023-07-07

**Authors:** Min Chen, Zhongyu Yan, Lu Han, Dandan Zhou, Yan Wang, Leiqing Pan, Kang Tu

**Affiliations:** 1 College of Food Science and Technology, Nanjing Agricultural University, Nanjing 210095, China; 2019208017@njau.edu.cn (M.C.); 2021808103@njau.edu.cn (Z.Y.); hanlu@njau.edu.cn (L.H.); 2020208021@stu.njau.edu.cn (Y.W.); pan_leiqing@njau.edu.cn (L.P.); 2 College of Light Industry and Food Engineering, Nanjing Forestry University, Nanjing 210037, China; dandanz@njfu.edu.cn

## Error in Figure

In the original publication, there is a mistake in Figure 1: A duplication error between 1B and 1C, which occurred due to the similarity of the images of the three nanoparticles. The corrected version of Figure 1 is presented below. The authors state that the scientific conclusions are unaffected. This correction has been approved by the Academic Editor. The original publication has also been updated.

## Figures and Tables

**Figure 1:**
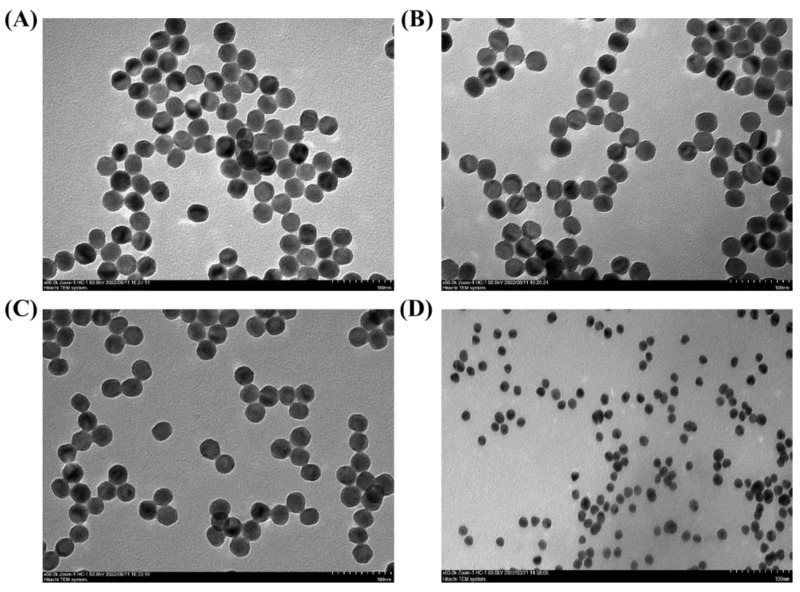
TEM image of OA−UCNPs (**A**), ADA−UCNPs (**B**), UCNPs−COOH (**C**), and GNPs (**D**).

